# Cold exposure and the cardiovascular system: from physiological adaptation to pathological risk

**DOI:** 10.3389/fphys.2025.1740919

**Published:** 2026-01-07

**Authors:** Yao Li, Jiawei Wu, Yinli Xu, Junbo Dong, Bo Xing, Yiwen Wang, Zijun Zhou, Boxuan Sun, Jiahui Li, Liming Yu, Huishan Wang

**Affiliations:** 1 College of Medicine and Biological Information Engineering, Northeastern University, Shenyang, China; 2 State Key Laboratory of Frigid Zone Cardiovascular Disease, Department of Cardiovascular Surgery, General Hospital of Northern Theater Command, Shenyang, China

**Keywords:** autonomic nervous system, brown adipose tissue, cardiovascular diseases, cold exposure, metabolic remodeling, molecular mechanisms

## Abstract

Cold stress is the therapeutic paradox of cardiovascular medicine: both an established environmental trigger of acute death and a physiological stimulus for powerful adaptation. In this review, we address this paradox critically. The response to cold challenge, which ranges from sympathetic activation and hemodynamic stress to adaptive brown adipose tissue recruitment and cardiac metabolic remodeling, which can be pathological or protective. We synthesize evidence from specific cardiovascular diseases, such as coronary disease and heart failure, and summarize molecular pathways of metabolic, inflammatory, and electrophysiological effects. Native adaptive mechanisms and their therapeutic potential as templates are also discussed. Through synthesis of these multi-faceted avenues, this article builds upon a theoretical basis to propose a mechanistic model for the seasonal regulation of CVD and to outline emerging, cold-based research avenues.

## 1 Introduction

Cardiovascular disease (CVD) is a major global cause of death and disability. While its etiology is multifactorial, with established risks including hypertension, diabetes, and cigarette smoking, there is a growing acknowledgment of the instrumental role played by environmental determinants, most notably ambient temperature (Fan J. F. et al., 2023; Alahmad et al., 2023). Epidemiological evidence firmly associates decreased temperatures with increased incidence and mortality of cardiovascular events. This risk is most apparent during cold winters or sudden cold episodes, when hospitalizations and CVD deaths acutely increase (Fan J. F. et al., 2023; Alahmad et al., 2023; Xu et al., 2022; Ma et al., 2020). This pattern is also evident from series analysis in Lanzhou, China, where both low and high temperatures trigger emergency room presentations for CVD (Ye et al., 2024). The worldwide importance of the topic is illustrated by a massive cross-city study across 567 cities, which demonstrated that cold extreme influences virtually all major CVD subtypes, including ischemic heart disease, heart failure, and stroke, and accounts for a substantial component of the incremental mortality burden worldwide (Alahmad et al., 2023). Most importantly, in China, this risk exhibits a strong north-south gradient: people living in the cold northern areas, have much higher cardiovascular and cerebrovascular disease mortality in comparison to the south, an effect explained by an interaction of diet and lowered ambient temperature (Wang M. et al., 2021).

Physiologically, cold stress is a powerful sympathomimetic stimulus, which triggers a cascade of acute cardiovascular responses including increased heart rate, peripheral vasoconstriction, and subsequent cardiac afterload augmentation (Ikäheimo, 2018; Drost et al., 2024; Prodel et al., 2022). These responses are probably synergistically combined to cause elevated myocardial oxygen demand (Prodel et al., 2022). The cold pressor test provides a clinically relevant illustration of such sympathetic stimulation, manifesting as increased heart rate and blood pressure, along with sexually dimorphic muscle sympathetic nerve activity neurovascular coupling (Coovadia et al., 2022). In healthy individuals, these responses are usually transient and compensatory (Miró et al., 2023). In patients with underlying CVD, such as CAD, heart failure, hypertension, or arrhythmias, the stressed circulation thus elicited may overstrain the heart’s already limited reserve (Ikäheimo, 2018; Miró et al., 2023; Cheng et al., 2021). This derangement of the reserve-mediated compensatory capacity may explain how acute coronary syndromes are precipitated, heart failure exacerbations are triggered, malignant arrhythmias are induced, or sudden cardiac death is caused (Ikäheimo, 2018; Miró et al., 2023; Cheng et al., 2021). For example, in CAD patients, the severity of coronary lesions, quantified using the SYNTAX score, correlates directly with cold exposure, and this is particularly true at night time (Li et al., 2024). Clinical observation also holds fast that cardiac workload during exercise in the cold is substantially higher compared to that in a thermoneutral environment, and this causes onset of myocardial ischemia at early phase in these patients (Ikäheimo, 2018). In addition to this augmented load, cold may also modulate hemorheological determinants, such as the plasma viscosity, and thus contribute to cardiovascular load (Ni et al., 2022).

**FIGURE 1:**
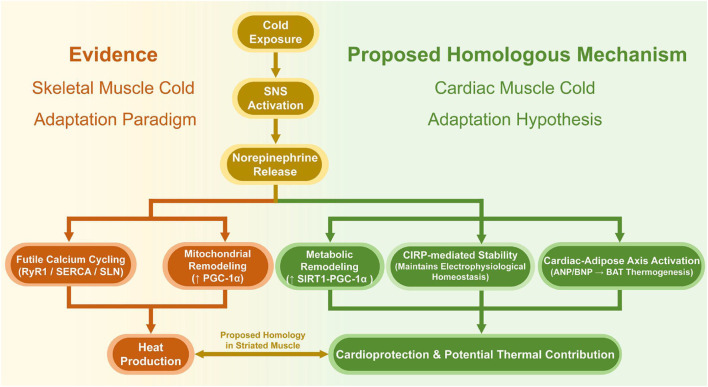
From established evidence to proposed hypothesis: a homologous framework for cold adaptation in striated muscle. (Left panel, Evidence in Skeletal Muscle): The established adaptive cascade to cold in skeletal muscle involves sympathetic nervous system (SNS) activation, norepinephrine (NE) release, and the subsequent induction of futile calcium cycling (via RyR1, SERCA, and SLN) and mitochondrial remodeling (upregulated PGC-1α), culminating in heat production. (Right panel, Proposed Homology in Cardiac Muscle): Based on the evolutionary and structural similarities between striated muscle types, we propose that cardiac muscle may employ homologous adaptive pathways. Cold-induced SNS activation and NE release could similarly drive metabolic remodeling (via the SIRT1-PGC-1α axis), promote electrophysiological homeostasis (mediated by CIRP), and activate the cardiac-adipose axis (ANP/BNP release stimulating BAT thermogenesis), collectively contributing to cardioprotection and potential systemic thermoregulation.

Of particular interest, however, is the extension of research beyond the acute, disease-inducing, effects of cold to investigate the adaptive, or even salutogenic remodeling of physiology induced by repeated or chronic exposure. Perhaps, the most important of these findings has been the activation of brown adipose tissue (BAT), such that thermogenesis and energy expenditure are possible, with resultant beneficial effects on whole-body glucose and lipid metabolism, and insulin sensitivity (Challa et al., 2024; Park et al., 2024; Christen et al., 2022; McNeill et al., 2020). Human research can also provide mechanistic information, demonstrating that acute cold stress results in changes in plasma levels of apolipoprotein M and sphingosine-1-phosphate, markers of brown fat metabolic activity (Borup et al., 2022). Furthermore, the adaptive responses to cold exposure in skeletal muscle, which shares the striated muscle classification with cardiac muscle, have been more extensively studied. Substantial evidence indicates that in birds, which lack brown adipose tissue (BAT), skeletal muscle serves as the primary site of non-shivering thermogenesis (NST). The core molecular mechanisms involve futile calcium cycling mediated by the ryanodine receptor (RyR1) and sarco/endoplasmic reticulum Ca^2+^-ATPase (SERCA), alongside SERCA-mediated thermogenesis regulated by sarcolipin (SLN) (Duchamp and Barré, 1993; Pani and Bal, 2022). Recent studies also support the fact that seasonal cold is enough to cause molecular adaptative responses in skeletal muscle even in adult birds, such as the upregulation of SERCA, RyR1, and SLN, and mitochondrial remodeling (Pani et al., 2023). Since there are basic similarities between cardiac and skeletal muscle in their structure, the elements that are involved in the contraction and those involved in the transport of the ions, it also follows that they are probably similar in terms of transcriptomic regulation alongside the adaptation to stresses. Therefore, based on the long-known information about cold adaptation of skeletal muscle, one can reasonably hypothesize that similar adaptive mechanisms may operate in the heart to manage cold stress. This theoretical framework, which connects the skeletal muscle evidence and the cardiac hypothesis, is shown in Figure 1 and provides a sound theoretical base, on which the deep exploration into the mechanism of cold effects on the cardiovascular system will be conducted in this review. Cold acclimation results in extensive metabolic reprogramming of even the heart itself and enhanced mitochondrial performance, which is at least partially supported by the upregulation of the SIRT1-PGC-1α pathway (Mohan et al., 2023a; Mohan et al., 2023b). Non-mammalian proteomic models, including zebrafish, have demonstrated a sequence of cardiac occurrences in cold acclimation initiated by the initial MAPK signals and cell remodeling before the mitochondrial electron chain components are downregulated further on (Shaf et al., 2024). Interestingly, chronic exposure to cold is noted to inhibit pathological remodeling of the heart through the regulation of autophagic events (Ruperez et al., 2022). The combination of these results provides a general view of the cold exposure in the form of a double-edged sword that has the potential to cause acute danger yet can also activate robust processes of metabolic regulation and cardioprotection. To elucidate this complex dualism, the aim of this review is to consolidate clinical practice, epidemiological data and experimental data to understand the conditions and mechanisms by which cold exposure is associated with physiological adaptation and/or disease risk. We will critically review the evidence for this “double-edged sword” effect with an eye to the creation of a deeper theoretical foundation and practical policy for seasonal prevention and control of CVD, high-risk patient management in the healthcare system, and to inform additional translational research in these domains. One final caveat is needed: while chronic exposure to sub-optimal temperatures (high and low) has been associated with death risk, presently available evidence still has a chance for bias that would need to be determined by higher-quality prospective studies in the future (Zafeiratou et al., 2021).

## 2 Cardiovascular physiological adjustments to acute cold exposure

Acute cold stress induces a cascade of cardiovascular adaptations by initiating the local vascular and neuroendocrine mechanisms. While these mechanisms are essential in core body temperature regulation, they concomitantly impose a significant burden on the cardiovascular system with particular risks for individuals with pre-existing cardiovascular disease.

### 2.1 Autonomic control and hemodynamic adaptations

The hemodynamic response to acute cold, typically studied using experiments such as the cold pressor test (CPT) or cold-water immersion, is defined by the sudden onset of sympatho-adrenal activity. The present evidence shows that the CPT is followed by a marked increase in heart rate, systolic and diastolic arterial pressure, and rate-pressure product (RPP) in healthy adults, unequivocal proof of increased myocardial oxygen demand (Drost et al., 2024; Bullock et al., 2023). However, this hemodynamic reactivity exhibits significant inter-individual variation. For instance, essential hypertensive subjects show blunted heart rate and diastolic blood pressure recovery and reactivity to parasympathetic activation after repeated CPT exposure *in vivo*, which assesses the body’s ability to adapt plastically to repeated cold stress (Bullock et al., 2023). This hemodynamic reaction, largely mediated by α1-adrenergic receptors, is markedly enhanced in postmenopausal women and is designed to emphasize the central importance of sex and age to vascular reactivity (Prodel et al., 2022). Beyond peripheral effects, such sympathetic activation produces a direct cardiac electrophysiological effect, including independent QTc interval prolongation and T-wave amplitude increase, changes only feebly correlated with systemic markers of stress like cortisol and therefore implying an alternative physiological origin (Drost et al., 2024). Furthermore, acute non-shivering cold stress also brings about deep changes in electrocardiographic parameters, including the P axis, QRS axis, and QT interval, although no direct correlation between these changes and BAT activity or plasma catecholamine levels has been reported (Raiko et al., 2021).

The autonomic nervous system also self-adjusts dynamically with exposure to cold. Following the initial sympathetic stimulation, short-term whole-body cold exposure (e.g., cryostimulation or cold-water immersion) has a seemingly paradoxical effect of increasing cardiac vagal tone. This is reflected in shifts in heart rate variability (HRV), characterized by increased high-frequency power (HF) and the root mean square of successive differences (RMSSD) and decreased low-frequency/high-frequency ratio (LF/HF) (Jdidi et al., 2024; Sun et al., 2024). Extensive inter-personal heterogeneity in thermoregulatory responses, such as “activity-thermoregulatory heat substitution”, in which certain individuals consistently exhibit lower energy expenditure during cold activity, is a feature linked with variables like body size and heart mass (Maloney and Careau, 2022). This parasympathetic stimulation in rebound can be partly explained by a “diving reflex” caused by cold and has a positive effect on cardiac autonomic regulation increased with long-term adaptive responses (e.g., repeated cold-water immersion), permitting post-exercise recovery (Malta et al., 2023). The complexity of autonomic control is also made clear by evidence that the threshold temperatures for the induction of sympathetic (via elevated mean arterial pressure) and cardiac parasympathetic (via elevated RMSSD and HF) activity are dissociable; in facial cooling models, a peak sympathetic response can be elicited at 7 °C, but a peak parasympathetic response can only be evoked by a more intense 0 °C stimulus (Gorini Pereira et al., 2024). At extreme cold temperatures (e.g., −5 °C to −15 °C), skin and blood pressure local temperature become the best indicator available to assess cold stress risk, with the initial phase of physiological stress on exposure endangering cognitive judgment and operating ability (Wu et al., 2021).

### 2.2 Blood pressure and vascular response

There is a long-established inverse relationship between blood pressure and environmental temperature, such that even minor decreases precipitate acute blood pressure elevations. Panel studies meticulously illustrate that in proportion to each decline in personal micro-environmental temperature by 1 °C, systolic and diastolic blood pressures in hypertensives rise by an average of 0.96 mmHg and 0.28 mmHg, respectively, within 24 h, a rise far steeper than in normotensive individuals (Xu Y. et al., 2024). Pressor effect is strongest 1–10 h after exposure and more during winter than summer (Xu Y. et al., 2024). Seasonal variation in blood pressure has a highly important effect on the epidemiology of hypertension, particularly in Asian populations, as cold-season increments may have the potential to drive increased incidence rates and complicate control (Park et al., 2020). High-population time-series research also corroborates this reverse correlation, quantifying that with each fall of 1 °C in temperature, risk of mortality and morbidity due to CVD is boosted by 1.6% and 1.2%, respectively (Fan J. F. et al., 2023). Winter disease threats’ dominance is also highlighted by research in Russian subarctic settlements, with the injurious effect of cold dominating that of heatwaves with some exceptions for subarctic settlements with extreme continental climate (Revich and Shaposhnikov, 2022). The speed and the duration of this effect are accounted for by ambulatory blood pressure monitoring, and the effect of ambient temperature on blood pressure has been found to start in the same exposure hour and last as long as 36 h (Fan P. et al., 2023). In addition to direct cardiovascular effects, the synergistic effect of cold and physical inactivity in experimental animal models results in a sharp rise in total serum cholesterol that can serve as an early indicator of systemic reprogramming of metabolism in the conditions of a cold environment (Shmakov et al., 2020).

Its mechanisms are revealed through central stimulation of the sympathetic system and complicated local vascular reactions. The principal role of cold-induced vasoconstriction is the minimization of cutaneous heat loss. Remarkably, dilatation caused by cooling as a new counter-regulatory mechanism was discovered in cutaneous small arteries. The protective dilatation, based on SK3-mediated endothelium-derived hyperpolarization and requiring enhanced communication via myoendothelial gap junctions, is a critical regulator for preventing pathological vasoconstriction for tissue damage (Chang et al., 2023; Flavahan and Flavahan, 2020). At the sensory level, transient receptor potential channel TRPM8 is implicated directly in behavioral cold environment thermoregulation, and enhanced expression following exposure may be a protective adaptive reaction (Thapa et al., 2021a). Downregulation of cold sensors like TRPM8 with age may lead to vasomotor dysfunction and enhance cold-induced blood pressure variability and tissue hypoperfusion (Thapa et al., 2021b). In addition to hypoxia of low pressure, cold stimulation was found to evoke relative preservation of thermoregulatory and cutaneous blood flow responses, as evidenced by increases forehead temperature and pulse with no net significant change, indicating hypoxia does not influence cold-evoked thermoregulation greatly (Shin et al., 2020). In addition, cold stress produces negative hemorheological effects, such as direct and persistent increase in plasma viscosity in conditions of cold climate, offering another significant pathophysiologic connection between cold and enhanced CVD risk (Ni et al., 2022).

### 2.3 Sympathetic activity and metabolic responses in skeletal muscle

In addition to its pronounced effects on the cardiovascular system and brown adipose tissue, cold exposure also modulates sympathetic nervous system (SNS) activity within skeletal muscle. However, its response pattern and tissue specificity differ significantly from those of classic thermogenic organs like BAT. Dulloo et al. conducted a systemic evaluation of the influence of the cold condition and diet on the SNS activity in the various rat tissues through direct measurement of the norepinephrine (NE) turnover, which is a functional index. They discovered that the acute cold exposure (4 °C) did raise NE turnover in skeletal muscles (e.g., gastrocnemius, soleus), indicating heightened sympathetic activity, however the extent of this increase was far less than that in the heart and in interscapular brown adipose tissue (Dulloo et al., 1988). The sympathetic response in skeletal muscle was relatively low even when the cold stimulus was sustained cold exposure over 1 week. In addition, the SNS activity in skeletal muscle was not affected by the dietary manipulations like fasting or feeding the organism on sucrose, unlike the heart and iBAT that are sensitive to such changes. The results of these studies support a high level of heterogeneity of sympathetic outflow and point to the fact that skeletal muscle is not a major locus of sympathetically mediated thermogenic activities to cold and that its role is rather minimal.

Nevertheless, skeletal muscle has the molecular apparatus that can mediate the calorigenic processes of catecholamines. As reviewed by Maslow and Vychuzhanova, catecholamines have the potential to trigger a non-shivering thermogenesis in skeletal muscle, in brown and white adipose tissue, likely through stimulation of β3-adrenergic receptors (β3-AR). It is linked to the process of upregulation of uncoupling proteins (e.g., UCP3), and increased lipid mobilization (Maslov and Vychuzhanova, 2016). Therefore, despite the net reduction in sympathetic stimulation of skeletal muscle, its intrinsic adrenergic signaling in bone, its metabolic reorganising capacity indicates the possibility of a range of control in long-term cold adaptation, and places it as a more than merely passive consumer of energy.

### 2.4 Role of mitochondrial plasticity in skeletal muscle during cold adaptation

In addition to the acute reactions that are orchestrated by the sympathetic nervous system, skeletal muscle plasticity in mitochondria that is triggered by a chronic cold treatment is core to maintaining its thermogenic potential. Research on high-altitude deer mice (a model exhibiting exceptional cold resistance) demonstrates that chronic cold exposure induces profound remodeling of mitochondrial function to enhance thermogenesis (Mahalingam et al., 2020). Key features of this remodeling include: (1) Enhanced Mitochondrial Uncoupling: This is expressed as increased leak respiration and reduced phosphorylation efficiency as well as OXPHOS coupling efficiency. A process similar to the role of UCP1 in BAT, which tries to uncouple substrate oxidation to ATP production is intended to maximize heat production; (2) Preference of Alterations in the Electron Transport Chain Complexes Preference: The reduction in the number of substrates oxidized by Complex II versus Complexes I + II indicates that the use of specific pathways to substrate oxidation may be rearranged in favor of other pathways.

Moreover, ultrastructural studies in mice further support this remodeling. Exposure to a non-thermoneutral condition, followed by even mild exposure to cold (16 °C), quickly starts a reorganization of skeletal muscle mitochondria that includes their increased abundance, morphological change to more elongated, tubular shapes and most importantly is a significant rise in cristae density. Since cristae are the main places of oxidative phosphorylation, their greater concentration literally means more energy generation and thermogenic possibilities of the mitochondria. These changes are even greater in extreme cold (4 °C). Notably, BAT and skeletal muscle coordinate mitochondrial restructuring during the cold adaptation process and indicates a possible cross-tissue communication mediator role by cytokines (e.g., FGF21 and IL-6) (Bal et al., 2017).

### 2.5 Thyroid hormones and the hypothalamic–pituitary–thyroid axis

In addition to autonomic and regional tissue adjustments, exposure to cold has global effects, affecting metabolism and cardiovascular activity by activating the hypothalamic-pituitary-thyroid (HPT) axis as one of the endocrine links between environmental and physiological or pathological phenotypes. Cold produces an effect of thyrotropin-releasing hormone (TRH) neurons in hypothalamic nucleus including the paraventricular nucleus, increasing plasma levels of thyroid hormone (TH), increasing basal metabolic rate and thermogenic capacity, and has extensive effects on cardiac structure and function (Zhang et al., 2018; Liu et al., 2025). Adaptive changes in HPT axis hormones have been observed in Antarctic expeditioners depending on ambient temperature, photoperiod, cold exposure time period, and level of stresses (Spinelli and Werner Júnior, 2022).

Direct cardioprotective effects of the thyroid hormones occur. Signs of protective pattern in chronic conditions of administration of thyroxine, p38 MAPK and PKC potentiation of activity modulatory effect on ischemic injury of myocardial cells and post-ischemic functional recovery are observed (Pantos et al., 2002). Hyperthyroid rat models, myocardial stunning induced by ischemia-reperfusion is blocked by TH that coordinates the activity of mitochondrial sodium-calcium exchanger and potassium channels, thus, increasing cardiac energetics and calcium homeostasis (Ragone et al., 2015). In addition to this, TH increases the activity of monolysocardiolipin acyltransferase in cardiac mitochondria, thereby facilitating cardiolipin remodeling and modifying the mitochondrial membrane structure and hemodynamics in the best way possible (Mutter et al., 2000). These results indicate that the activation of the HPT axis during cold can increase cardiac tolerance to cold stress and ischemic stress indicating that cardiac tolerance to cold stress and ischemic stress works better in cold conditions.

Whole-body energy metabolic processes are also coordinated by activation of the HPT axis which has a secondary effect on cardiovascular load. Cold exposure activates TH via HPT axis, which subsequently increases brown adipose tissue and skeletal muscle mitochondrial biogenesis with uncoupling thermogenesis, regulated by β-adrenergic/cAMP/PKA/p38 MAPK/CREB signaling pathway and PGC-1 increased expression (Hohenauer et al., 2025). TH being a key signaling molecule, integrates environmental signals including nutrition, temperature, and photoperiod to coordinate seasonal metabolic, thermogenic and reproductive seasonal processes (Liu et al., 2025). In this way, the HPT axis forms an integral endocrine process of energy redistribution and increased body metabolic rate in adapting to cold.

Still, this adaptive axis is also of a two-sided nature. With prolonged or chronic cold exposure, prolonged HPT axis activation can cause excessive metabolic stimulation and elevated myocardial workload, which can put further stress on the myocardial oxygen demand and promote maladaptive remodeling, especially when pre-existing heart disease is present. Furthermore, the number and severity of thyroid disorders are also seasonal, meaning that environmental influences may affect the tendency of the HPT axis in disease pathogenesis (Liu et al., 2025).

## 3 Cold exposure and cardiovascular pathophysiology: mechanisms and therapeutic translation

The significance of cold exposure for cardiovascular outcomes is not explained in terms of single, disease-specific effects, and rather as representations of a restricted number of underlying pathophysiological pathways. The three pathways that include acute hemodynamic stress, chronic maladaptive remodeling, and electrophysiological instability are triggered to diverse magnitudes in various cardiovascular conditions. The clear explanation of these common mechanisms can offer a better framework of risks stratification and can offer new therapeutic prospects to take advantage of adaptive responses to cold.

### 3.1 Acute hemodynamic stress and ischemic events

A rapid mismatch between myocardial oxygen supply and demand is central to the pathogenesis of Acute Myocardial Infarction (AMI) and Acute Coronary Syndromes (ACS) and the actual failure of the myocardium in the heart underlying Acute Aortic Dissection (AAD), shares a common focus. The exposure to cold triggers this severe imbalance in a complex pathophysiology. The strong cold-induced sympathetic stimulus has a pronounced stimulating effect on the heart rate and blood pressure, changes of the myocardial contractility which results in substantially high rate-pressure product (RPP) and, as a result, in an increase in myocardial oxygen demand (Ikäheimo, 2018; Valtonen et al., 2022). This increased demand may immediately exceed a fixed or limited supply in the presence of coronary stenosis leading to ischemia or infarction. Meanwhile, cold inhibits oxygen delivery by causing coronary vasospasm and decreasing coronary blood flow (Ikäheimo, 2018), and promotes a pro-thrombotic environment by increasing blood viscosity and high plasma concentrations of fibrinogen contributes to the support of the risk of pro-occlusive coronary thrombosis (Ni et al., 2022). Moreover, the same hemodynamic factors that manifest in drastically elevated blood pressure and high ejection velocity of the left ventricle exert enormous shear stress on the aortic wall which is a major precipitant of AAD in predisposed persons (Chen et al., 2022; Yu et al., 2021; Hu et al., 2023; Sadamatsu et al., 2020). This danger is further enhanced by the high rates of change in temperature, which is believed to chronically harbor endothelial functioning and unbalances atherosclerotic plaques (Chen et al., 2022; Ma et al., 2021). The treatment problem, consequently, is to reduce this acute danger. Such potential is not in the application of acute cold but in the application of chronic and controlled cold adaptation to create a physiological condition of resistance to such stressors. Middle-range models have revealed that moderate cold acclimation has the capability of providing potent cardioprotection that mitigates myocardial infarct size and modulating catecholamine-saturates β-adrenergic receptors (Marvanova et al., 2023). This implies that, in the long-term, a well-modulated protocol of intermittent cold therapy may re-normalize the sympathetic response, and enhance vascular reactivity, so that the pathological hemodynamic effect of acute cold stressor is prevented. The best way forward would therefore be to conduct research on the best conditioning protocol of cold conditioning in high risk patients to ensure that the percentage of cold induced acute events is reduced.

### 3.2 Chronic load and maladaptive remodeling

A central phenomenon leading to the exacerbation and progression of chronic diseases like Heart Failure (HF) and Hypertension is a long-term mechanism of chronic hemodynamic and metabolic stress, with stress-induced by cold continually promoting a vicious cycle of pathological adaptation. Pathophysiological underpinnings of this pathway include many, which frequently interact, aspects. To begin with, cold exposure causes systemic vasoconstriction and places an excessive burden on the heart in terms of afterload; in the case of an already compromised heart already working within the confines of its compensatory reserves, this hemodynamic change easily falls into clinical decompensation and hospitalization (Miró et al., 2023; Escolar et al., 2019). More so, hypertensive people have exaggerated pressor effect of cold which is enhanced during winter (Xu Y. et al., 2024; Zhang et al., 2023); this recurring stressor has the capacity to directly ideate left ventricular hypertrophy and progressive vascular damages. A particularly vicious cycle exists between HF and cold via the Heart Failure-BAT axis. HF causes dysfunction of the BAT in turn disrupting choline metabolism causing the increase in the levels of the cardiotoxic metabolite TMAO. TMAO inhibits the mitochondrial activity of the myocardium and empties the energy resources, which additionally deteriorates the cardiac dysfunctions (Yoshida et al., 2022). This metabolic dysfunction-chronic overload loop presents a promising therapeutic target. Of particular interest is the possibility of selective BAT stimulation. As the BAT dysfunction is a symptom of the HF, the strategies aimed at the recovery of its activity (pharmacologically or by the means of controlled and mild cold exposure) might benefit the normal body metabolism and decrease the production of TMAO. In the case of hypertension, when Angiotensin-(1–7) was found to induce WAT browning and thermogenesis (Vargas-Castillo et al., 2020), it opens up a new, conceptually conceptualized cold-mimetic pathway in metabolism of blood pressure regulation. This therefore makes the clinical trials designable in order to examine whether the activation of the non-shivering thermogenesis through the safe cold exposure activities or even the use of pharmaceutical agents can enhance the metabolic conditions of patients with stable HF or hypertension and inhibit the onset of the disease.

### 3.3 Electrophysiological instability and arrhythmogenesis

Cold stress has a direct pathogenic relationship with the myocardial electrophysiological system, and it shocks the mechanism of electrical homeostasis, creating a substrate of arrhythmias and a high risk of Sudden Cardiac Death (SCD). Several mechanisms underlie this pathological pathway. Firstly, cold pressor test results in the long-term repolarization, which is reflected by the prolongation of QTc interval and higher T-wave amplitude, which suggests the increased dispersion of ventricular repolarization and the formation of a substrate depolarization re-entrant arrhythmia (ventricular tachycardia and ventricular fibrillation) (Drost et al., 2024). Secondly, during individuals who are susceptible and with underlying channelopathies such as long QT Syndrome (LQTS), cold exposure can elicit an unusually steep QT/heart rate relationship, which puts them at an amazing risk of life-threatening events at low heart rates (Takahashi et al., 2020). Thirdly, the dysregulation of protective molecular stabilizers is also crucial; particularly, deficiency of the cold-inducible RNA-binding protein (CIRP) as an important physiological stabilizer that causes a pro-arrhythmic condition by activating potassium channels, quickening repolarization, shortening the action potential duration, and consequently predisposing to arrhythmia like atrial fibrillation (Xie et al., 2020a; Xie et al., 2020b). Consequently, prevention and stabilization is cautioned as the therapeutic emphasis of this pathway instead of the use of cold as an intervention. The importance of CIRP as the key new target of anti-arrhythmic therapy is highlighted by the fact that the approach to preserving or optimizing its activity may help prevent electrophysiological destabilization of the body under cold stress. Beyond, it is possible that additional processes by which intrinsic electrical resilience occurs can identify new processes by investigating the possibility that long-term adaptation to cold (such as in experienced cold-water swimmers) can increase the process of endogenous CIRP and other stabilizing proteins. For clinical practice, these observations demonstrate how important patient education and strict protection, indicating that individuals with known arrhythmogenic conditions should avoid experiencing acute cold strictly.

### 3.4 Winter milieu complex: confounding factors on CVD risk in cold

The pathophysiological pathways outlined above, acute hemodynamic stress, chronic maladaptive remodeling, and electrophysiological instability, have a unified mechanistic explanation of how cold exposure poses a direct challenge to the cardiovascular system. Nevertheless, the severe winter spike of CVD morbidity and mortality witnessed in epidemiology studies demonstrates that in nature the risk environment is never typically determined solely by a single climatic influence (Wang et al., 2020). Cold stress typically occurs within a complex seasonal environment in which several confounding factors come together and may act synergistically with the direct impacts of low temperature (Pettersson et al., 2020). As the epidemiological and pathophysiological data indicate that cold exposure is a direct cause of the acute CVD event (Borchert et al., 2023; Saucy et al., 2021) it is important to note that cold stress can regularly take place in a very complicated seasonal context, which is riddled with a range of confounding factors. These aspects may either act alone, or interact with each other in a synergistic manner with the cardiovascular burden of cold. To start with, there is an increase in respiratory infections (e.g., influenza) in the winter that may trigger an acute cardiovascular event due to systemic inflammation, augmented metabolic rate, and pro-thrombotic actions (Achebak et al., 2024). Second, seasonal air pollution, especially in areas that use fossil fuel to heat buildings, may further increase endothelial dysfunction and oxidative stress which may combine with cold to increase risk to the heart (Rivera-Caravaca et al., 2020). In addition, cold-month changes like decreased physical activity and changes to lower-energy food intake may result into gaining weight and unhealthy changes in blood pressure, and thus a change in baseline risk profile of an individual (Wang M. et al., 2021). Finally, pharmacological factors should be taken into account as, e.g., β-blockers can attenuate the tachycardic response to cold that might lead to a lack of efficient thermoregulation and one of the physiological compensation systems may be concealed (Tang et al., 2023). Therefore, the CVD morbidity and mortality winter peak observed probably represents only an interaction between the direct effect of the cold environment and these concomitant seasonal difficulties, and thus highlights the necessity of combined public health action to handle this syndemic of environmental and behavioral hazards.

## 4 Cellular and molecular basis of cold’s action on the cardiovascular system

**FIGURE 2:**
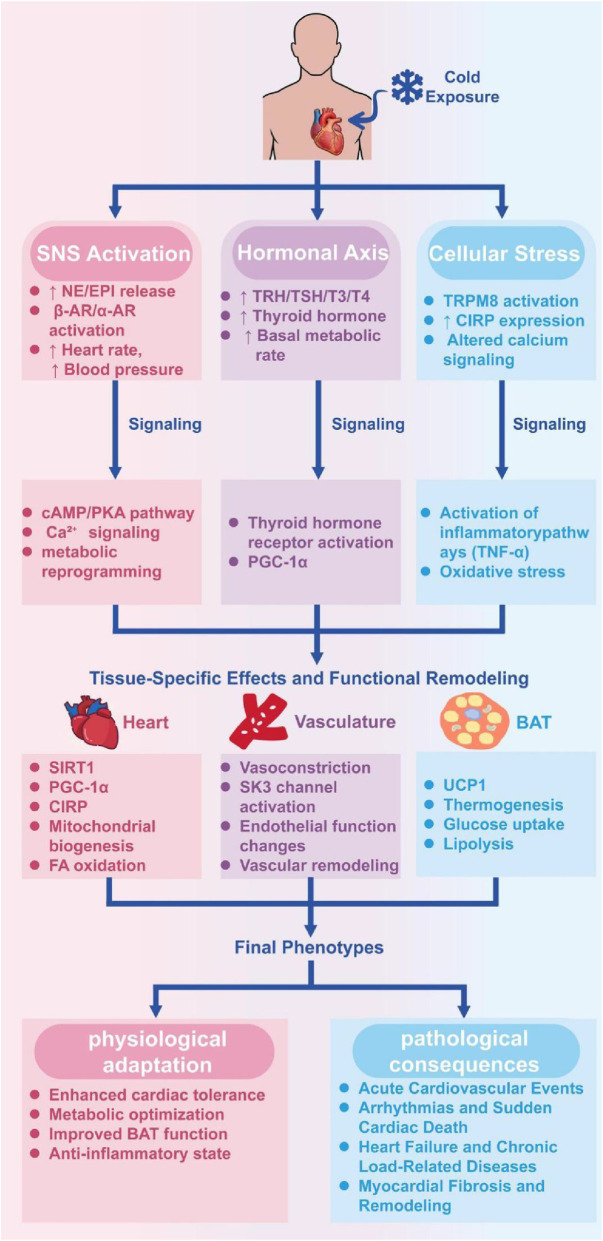
Cold Exposure Signaling cascades: The mechanistic foundations of dual cardiovascular outcomes. The following schematic represents the major molecular pathways involved following cold exposure which results in either physiological adaptation (left) or pathological effects (right). Peripheral thermoreceptors (e.g., TRPM8) and central thermoregulatory centers initially receive cold stimuli and are sensed by three major response axes: (1) Sympathetic Nervous System (SNS) activation releasing norepinephrine (NE); (2) Hypothalamic-Pituitary-Thyroid (HPT) axis activation that raises thyroid hormone (TH) levels; and (3) local cellular-level stress responses such as cold-inducible RNA-binding protein (CIRP) up These major cues diverge on tissue-specific effectors: in the heart they generate protective metabolic reprogramming by SIRT1-PGC-1α, and electrophysiological stabilization by CIRP; in vasculature they regulate vasoconstriction/vasodilation; in brown adipose tissue (BAT) they induce thermogenesis by UCP1. Adaptive effects (left) are improved cardiac tolerance, metabolic optimization, and communication between cardiac-adipose axis. Nonetheless, when abnormal and/or acute, and/or found in vulnerable hosts, these pathways rather sustain pathological cascades causing acute hemodynamic stress, chronic maladaptative remodeling, electrophysiological instability, inflammation and fibrosis. The final phenotype results as a combination of cold exposure variables (intensity, duration, pattern) and host variables (age, preexisting disease, genetic background).

The negative clinical consequences of cold summarized in the preceding chapter are due to generalized molecular and cellular changes. In addition to the integrated whole body neurohumoral responses, we address in this section, the basic underlying mechanisms: metabolic, electrophysiologic and inflammatory that determine the outcome of cold stress on the cardiovascular system by dictating the fate of the cardiovascular system to adaptation or disease. We discuss how these pathways,in concert with essential inter-organ communication, determine whether adaptation or disease will occur. The complex interplay of these signaling pathways, which ultimately dictate the adaptive or maladaptive outcome, is summarized in Figure 2.

### 4.1 Cardiac metabolic remodeling

As a high energy requiring organ, the heart is subjected to changes in its metabolic program following cold exposure. The short term would seem to be adaptive to cold stress by upregulating the signaling pathway SIRT1-PGC-1α, which promotes mitochondrial biogenesis and function, and increases the capacity for fatty acid oxidation, thereby enhancing cardiac cold tolerance (Mohan et al., 2023a; Mohan et al., 2023b). This adaptive response was recently extended to include intermittent cold stress upregulating cardiac SIRT3, reducing mitochondrial protein acetylation, and enhancing antioxidant defenses via MnSOD, collectively improving mitochondrial metabolic function (Mohan et al., 2023a). Mitochondrial physiological changes with regards to hypothermia are complex; they extend the mitochondria and they inhibit oxygen consumption by inhibiting Drp1, a process because of the inactivation of TRPV1 channels (Taylor et al., 2021). Notably, a metabolic defense of decoupling reactive oxygen species production, respiration, and Ca^2+^ dynamics in mitochondria in heart occurs under hypothermic conditions. The protective effects associated with mild degrees of hypothermia are partly due to blockage of reverse electron transfer as well as blockage of MCU facilitated Ca^2+^ uptake (Stevic et al., 2022).

A distinction between adaptation and pathology is narrow though. The chronic exposure to cold may force the heart into maladaptive remodeling phenotype. Such exposure induces cardiomyocyte enlargement, ANP/BNP hypertrophy marker gene expression and downregulation of fatty acid oxidation genes, which is phenotypically similar to pathologic hypertrophy (Ruperez et al., 2022; Kolleritsch et al., 2023). The process is plastic and reversible. Cardiac hypertrophy caused by cold reverts with re-establishment of thermoneutrality by the induction of autophagy (Ruperez et al., 2022). Another factor has been indicated to be autophagy which plays a critical role in cardiac structural remodeling (Ruperez et al., 2022).

The ultimate solution is: The therapeutic benefit of this route is restated in ischemia-reperfusion conditions where intensive cardioprotection with hypothermia is brought about by augmented autophagic level and mite-related quality (Marek-Iannucci et al., 2019). The path of cardiac energy substrate is also complex to control. May be phosphorylated by PKA: In basal conditions, the lipid droplet-bound protein Perilipin 5 (Plin5) acts as a context-specific rheostat: the CGI-58 sequestration protein Plin5 inhibits ATGL under normal conditions and lipolysis (Zhang et al., 2022). However, when exposed to cold stimuli, Plin5 is phosphorylated by PKA, which removes the inhibition on ATGL to increase the mobilization of fatty acids to cardiac oxidation to support the energy homeostasis in the body Such responses can even be preprogrammed in advance, e.g., the exposure of maternal cold triggers the offspring hypertension by the dysfunctioning of hypothalamus and hyperactivation of sympathetic bisynaptic responses (Chen et al., 2019).

**FIGURE 3:**
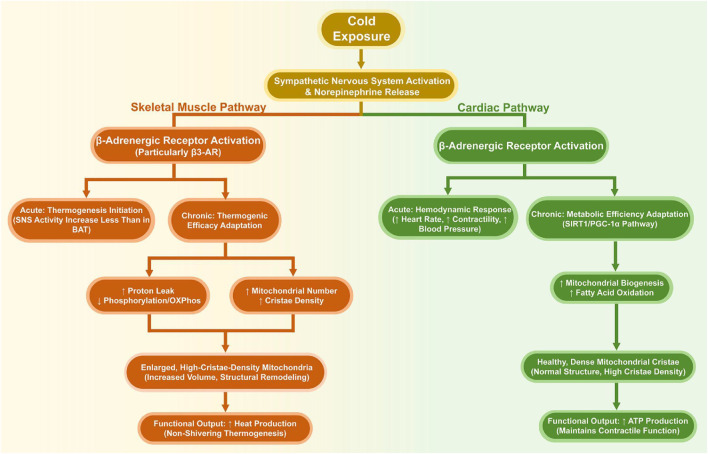
Divergent Adaptive Strategies of Cardiac and Skeletal Muscle to Cold Exposure. This is a comparative figure that shows major disparities in the heart and skeletal muscle response to and adaptation during cold stress. Although they are both striated muscles that both are subjected to sympathetic stimulation, the adaptations of the two differ greatly because they have different main physiological roles. Heart competitions metabolic efficiency and contractile function (e.g., through the SIRT1-PGC-1 actions), skeletal muscle remodels to increase thermogenic capacity (e.g., by mitochondrial uncoupling and cristae biogenesis), serving as a distributed warmer to the rest of the system.

Comparative Insight with Skeletal Muscle: The primary point of divergence is evident on the basis of the detailed above processes (Sections 2.4, 4.1). Although cold stress and catecholaminergic drive are commonly applied, heart and skeletal muscle are characterized by strongly different mitochondrial related adaptation strategies as their physiological functions are different. As a distributed thermogenic organ, skeletal muscle shows adaptations that favour thermogenic performance. This is defined by a high uncoupling propensity alongside systemic augmentation in cristae density both of which contribute to systemic non-shivering thermogenesis. On the other hand, the heart as a constantly operating pump focuses its mitochondrial adaptation on the preservation or regulation of the efficiency of the metabolism. This maintains a permanent ATP production to maintain the contractile ability during hemodynamic stress caused by coldness. Upregulation of mitochondrial biogenesis and fatty acids oxidizing potential through the SIRT1-PGC-1α pathway in the heart is, most importantly, a metabolic, but not active, uncoupling thermogenesis. It is through this functional specialization that the systemic division of labor is effectively captured, skeletal muscle as distributed heaters and the heart as a high-efficiency perpetual motion machine where harmonic function is confined to supporting its own functionality, and has no role to play in thermogenesis. It is this inherent weakness that, in part, could position why the heart would be readily susceptible of cold-induced injuries even though it is well set to withstand plastic reactions. The comparing systematic approach on cold adaptation of cardiac and skeletal muscle tissues is summarized by Figure 3 by showing the divergent sympathetic response and the divergent mitochondrial response based on the different physiological priorities.

### 4.2 Ion channels and electrophysiological remodeling

Cold exposure has a direct negative effect on the electrophysiological integrity of the heart, promoting arrhythmogenesis. Cold-inducible RNA-binding protein (CIRP) is one of the electrical stability proteins. It has a protective effect, localizing and stabilizing phosphodiesterases PDE4B and PDE4D mRNA and consequently modulates cAMP levels in the sinoatrial node cells and sustaining the guard against an excessive increase in heart rate in case of being burdened with the conditions of the β-adrenergic stimulation (Xie et al., 2020a). CIRP itself is controlled in a complex fashion, with hypothermia in non-hibernating mice altering the splicing of CIRBP transcripts to favor shorter forms that encode functional protein, demonstrating that splicing control is a conserved regulatory mechanism (Horii et al., 2019). Conversely, lack of CIRP fosters a pro-arrhythmic state, upregulating potassium channels (Kv1.5 and Kv4.2/4.3, for example) to accelerate repolarization, shorten the action potential duration, and enhance vulnerability to atrial fibrillation (Xie et al., 2020b). Besides myocyte-autonomous mechanisms, voltage-gated potassium channel KCNQ1 is also a sexually dimorphic sensor of moderate cold, coordinating with TRPM8 to control cold avoidance behaviors in male mice (Kiper et al., 2024). Additionally, the gap junction protein Connexin 43 (Cx43), which is essential for intercellular electrical coupling, is also remodeling in cold adaptation. Cold acclimation in spontaneously hypertensive rats improves Cx43 and its phosphorylated isoform (p-Cx43Ser368) expression, improves its distribution, and suppresses the TGF-β1/SMAD pro-fibrotic pathway in the meantime, revealing a mechanism of cold-induced electrophysiological protection (Andelova et al., 2022).

### 4.3 Inflammation, fibrosis, and cell death

Chronic exposure to cold establishes a pro-inflammatory and pro-fibrotic environment, predisposing to pathological remodeling. In a rat model of combined high-altitude and cold (HAC) exposure and hemorrhagic shock, cold exacerbated cardiac fibrosis, which was associated with upregulation of inflammatory cytokines (TNF-α, IFN-γ) and downregulation of anti-apoptotic protein Mcl-1 (Xu J. et al., 2024). This is in part orchestrated by the TGF-β/SMAD signaling pathway, inhibition of which is for instance, by 2,5-Dimethylcelecoxib (DM-C) via activation of GSK-3 and subsequent inhibition of Smad2/3 phosphorylation, effectively decreases cardiac fibrosis following injury (Ikushima et al., 2022). The translational promise of cold-induced inflammation is seen in findings that elevated serum levels of extracellular CIRP (eCIRP) in patients post-cardiac arrest are linked with higher systemic inflammation, disease severity, and are predictive of adverse 28-day outcomes (Wang L. et al., 2021). Extracellular matrix (ECM) integrity is not only critical in the heart but also in fat tissue, where defective ECM remodeling in BAT is linked to fibrotic inflammation and defective diet-induced thermogenesis (Pellegrinelli et al., 2023).

In addition to classical mechanisms, novel cell death forms contribute to cold-induced damage. Evidence is provided for the role of ferroptosis, which is a cell death induced by lipid peroxidation, in cold-stressed hearts. Remarkably, Beclin1 haploinsufficiency significantly improves cold-induced cardiac dysfunction and metabolic perturbations by inhibiting ferroptosis and mitochondrial injury (Yin et al., 2020). Metabolic disorder in cold stress is uncovered in plasma dynamic profiles and TAG profiles, in which the early reduction of unsaturated TAGs to provide thermogenesis is followed by the rise of liver-derived, polyunsaturated-enriched TAGs accompanying concurrent adipose tissue lipolysis, a process ATGL-dependent (Straat et al., 2022). Dietary treatment, for example, full-fat rice bran supplement, has been demonstrated to have the potential to circumvent this damage by augmenting cardiac antioxidant defense mechanisms (e.g., via Nrf2/HO-1 induction) and simultaneously inhibiting mitophagy, ferroptosis, and pyroptosis (Sun et al., 2023).

### 4.4 Brown adipose tissue (BAT) and the cardiac-adipose axis

The “Cardiac-Adipose Axis” new paradigm foresees an intrinsic, bidirectional cross-talk between the heart and adipose tissues, and in particular BAT, whose functional activity dictates on a large scale systemic metabolic and cardiovascular homeostasis (Tang et al., 2023; Lu et al., 2023). BAT activation is the substrate of non-shivering thermogenesis. On cold exposure, BAT β3-adrenergic receptors are stimulated by sympathetic release of norepinephrine, and this results in UCP1-mediated heat production along with co-uptake of fatty acids and glucose with beneficial effects on total-body metabolism (Challa et al., 2024; Christen et al., 2022; McNeill et al., 2020). Advanced multilayered regulation of this activation is also underway such as with the presence of intrinsic brakes such as a cold-activated truncated version of adenylate cyclase 3 (AC3-AT) that suppresses the formation of cAMP to prevent over-activation of BAT and exhaustion of energy (Khani et al., 2024). Regulation is also observed in mitochondrial dynamics, as the PACT RNA-binding protein inhibits mitochondrial biogenesis by activating miR-181c maturation (Dogan et al., 2022), and in inter-organelle contact, wherein the lipid droplet protein PLIN5 recruits mitochondrial FATP4 to augment fatty acid transport and oxidation during stress (Miner et al., 2023). Other layers include PEMT regulation of UCP1 splicing in a non-cell-autonomous fashion (Johnson et al., 2020) and the unexpected function of myoglobin (MB) in BAT, whose lipid-binding activity induces lipolysis and mitochondrial respiration after adrenergic stimulation (Christen et al., 2022). Some others, like the cold-regulated ubiquilins, are not required for BAT protein homeostasis and thermogenesis (Muley et al., 2021).

The “axis” is extremely bidirectional. Cardiac-secreted hormones ANP and BNP exert direct actions to activate BAT and browning of white adipose tissue, enhancing total energy expenditure (Lu et al., 2023; Kashiwagi et al., 2020). Conversely, BAT dysregulation in heart failure models leads to diminished thermogenesis and, through disrupted choline metabolism, elevates the cardiotoxic metabolite TMAO, which inhibits cardiac mitochondrial function and exacerbates dysfunction (Yoshida et al., 2022). This multiplicity is further attested by sex-differential behavior; diet-induced obese female mice cardiac-specific overexpression of βARKct increased insulin sensitivity but paradoxically caused deleterious cardiac remodeling and suppressed BAT thermogenesis (Manaserh et al., 2023). Smad4-mediated endothelial cell angiogenesis also promotes white adipose tissue through the proliferation of beige adipocyte progenitors (Wang et al., 2023). The delicate balance of nitroso-redox homeostasis in BAT, regulated by ADH5-catalyzed S-denitrosylation of proteins (conserved in HSF1) is required for UCP1-dependent thermogenesis and a potential target (Sebag et al., 2021). Together, these lines of investigation place BAT on the pedestal from coarse heater to an important regulatory center for cardiovascular health.

## 5 Harness cold adaptation for cardiovascular defense

Because the massive cardiovascular indebtedness of cold climate has become firmly established, application of best counterstrategies is necessary. The literature in this section shifts from solution assessment back to problem recognition, noting the manner in which we are able to initiate the body’s machinery of adaptation prior to a cold insult, through preconditioning, behavioral adaptation, and pharmacologic exploration, so that we can maximize cold tolerance and minimize risk of CVD.

### 5.1 Preconditioning and cross-adaptation: en route to a tolerogenic phenomenotype

The process of hormesis, where enhanced tolerance to a greater stressor is elicited by a smaller stressor, is graphically demonstrated by repeated chilling. There is now compelling evidence in animal models that acute MCA of short duration provide robust cardioprotection, reducing myocardial infarct size significantly and potentiating mitochondrial resistance to calcium overloading (Marvanova et al., 2023). The mechanisms are sophisticated and include protective desensitization of β1/β2-adrenergic receptors to catecholamine-induced cardiac load while inducing the simultaneous stimulation of the pro-survival β3-AR/PKG/AMPK pathway and a healthy serum immunomodulatory cytokine profile (Marvanova et al., 2023). Moreover, this therapeutic approach is not associated with adverse effects such as hypertension or cardiac hypertrophy (Marvanova et al., 2023).

This adaptive response is found to exhibit cross-talk with other stress signals, such as exercise- and hypoxia-induced signals. Alternating cold exposure, for instance, activates the principal regulators of mitochondrial biogenesis and function (e.g., PGC-1α, NRF-1) via PKA and SIRT3 signaling, thereby augmenting the metabolic and antioxidant reserve of the heart as well as its overall stress resistance (Mohan et al., 2023a). In keeping with the concept of common adaptive pathways, mouse models illustrate that cold exposure modifies thermoregulatory parameters (e.g., lowered skin, elevated core temperature), yet induces a gene expression pattern of mitochondrial biogenesis and mitophagy that is remarkably similar to exercise in room temperature (Opichka et al., 2019). Similarly, combined intermittent hypobaric hypoxia plus cold exposure can produce adaptive physiological adaptations like skeletal muscle angiogenesis (Santocildes et al., 2021). However, as the recent review indicates, coordinated thermoregulatory and cardiovascular control of humans with combined hypoxia and cold remains a major and as-yet-unresolved frontier (Mugele et al., 2021). Considered collectively, this work implies that controlled exposure to mild stress can be used as a non-pharmacologic intervention for improving cardiovascular health more broadly.

### 5.2 Behavioral and pharmacological interventions

Building on the concept of cold adaptation, a number of voluntary cold exposure modalities have been investigated for their cardiometabolic protective benefits and adverse dangers. As systematically compared in Table 1, the exposures of Cold Pressor Test (CPT), Cold-Water Immersion (CWI), Whole-Body Cryostimulation (WBC), and controlled ambient cold exposure each induce distinct physiological responses and have specific uses and limitations. This comparative framework is of value in selecting appropriate interventions on an individual’s health status and desired outcomes, to be elaborated in greater detail in the presentation of behavioral and pharmacological interventions.

**TABLE 1:** Cardiovascular effects and potential applications of different cold exposure modalities.

Intervention modality	Primary physiological effects	Potential benefits	Potential risks/considerations	Target population/research stage
Cold Pressor Test (CPT) (e.g., hand/foot immersion in ice water)	Acute, intense sympathetic activation; sharp increases in blood pressure and heart rate; elevated cardiac afterload (Drost et al., 2024; Prodel et al., 2022; Bullock et al., 2023)	Useful for assessing cardiovascular stress reactivity and autonomic function; identifies high-risk responses in hypertensive or CAD patients (Ikäheimo, 2018; Han et al., 2022)	Risk of triggering acute events in individuals with pre-existing CVD (e.g., uncontrolled hypertension, unstable angina) (Ikäheimo, 2018; Han et al., 2022)	Diagnostic/Research tool; suitable for clinical screening and basic research under medical supervision
Cold-Water Immersion (CWI) (Whole-body or partial)	Initial sympathetic storm, potentially followed by enhanced parasympathetic activity; decreased core/skin temperature; increased vasoconstriction (Jdidi et al., 2024; Sun et al., 2024; Malta et al., 2023; Eimonte et al., 2021)	May improve post-exercise recovery (characterized by reduced inflammation and muscle soreness); chronic or intermittent use may enhance cardiac vagal tone (↑HRV) (Malta et al., 2023)	Acute cardiovascular stress (Jdidi et al., 2024); can increase cardiac workload in CAD patients during exercise (Valtonen et al., 2022); risk of vasovagal reaction, hypothermia, and arrhythmias (especially with concurrent SNS/PNS activity) (Barwood et al., 2024; Lundell et al., 2020); elevates stress hormones and transiently alters immune markers (Eimonte et al., 2021)	Healthy individuals, athletes for recovery (Malta et al., 2023; Russell et al., 2021); explored in clinical research for metabolic benefits (Russell et al., 2021). Extreme caution in CVD patients (Valtonen et al., 2022)
Whole-Body Cryostimulation (WBC)	Brief but very intense stimulation of skin cold receptors; marked sympathetic activation, often followed by a parasympathetic rebound; increases mean BP and cardiac parasympathetic indices (RMSSD, HF) (Jdidi et al., 2024; Theurot et al., 2021)	May enhance cognitive inhibition performance, partly mediated by increased parasympathetic tone and cerebral oxygenation (effects may be sex-specific) (Theurot et al., 2021); may reduce inflammation (Jdidi et al., 2024)	Similar to CWI but more superficial stimulus; high cost; risk of cold burns; may induce bronchospasm in asthmatics	Primarily used in healthy individuals and athletes (Theurot et al., 2021); clinical research in conditions like fibromyalgia and rheumatoid arthritis. Evidence in CVD patients is insufficient
Ambient Cold Exposure (Natural or artificial cold environment)	Sustained, low-to-moderate intensity sympathetic activation; chronic increase in energy expenditure, blood pressure, and cardiac load to maintain thermostasis (Fan et al., 2023a; Xu et al., 2024a; Chen et al., 2021). Can induce pathological cardiac remodeling in mice (Ruperez et al., 2022; Portes et al., 2023)	Adaptive exposure (e.g., intermittent cold acclimation) can induce BAT activation, improve glucose/lipid metabolism (Challa et al., 2024; Park et al., 2024; McNeill et al., 2020), and may confer cardioprotection (e.g., reduced infarct size, improved mitochondrial function) via mechanisms like β-AR signaling remodeling and SIRT-PGC-1α pathway activation (Mohan et al., 2023a; Marvanova et al., 2023)	Non-adaptive exposure (e.g., winter cold, cold spells) is a well-established trigger for cardiovascular mortality and morbidity (AMI, HF, stroke, aortic dissection) (Fan et al., 2023a; Alahmad et al., 2023; Miró et al., 2023; Cheng et al., 2021; Chen et al., 2022); associated with increased plasma viscosity (Ni et al., 2022) and autonomic dysregulation (Chen et al., 2021)	Public health measures for seasonal protection are crucial (Achebak et al., 2024; Leuillier et al., 2023; Zhang et al., 2021a); intermittent cold acclimation as a proactive health strategy is still in experimental stages (Mohan et al., 2023a; Marvanova et al., 2023), not yet a clinical recommendation

#### 5.2.1 Temperature control and public health

Most simply, individual temperature control is a direct and very effective defense. Estimates by modeling show that in countries like Australia, simple improvements in domestic insulation levels to raise indoor temperature in cold homes from 16 °C to 20 °C could bring substantial reductions in CVD burden, with benefits equal to or greater than those of long-term dietary or lifestyle interventions among high-risk adults (Singh et al., 2022).Public health connotation is clearly revealed by Hong Kong time series data showing five times rise in cardiorespiratory cold attributable years of life lost in 2000–2007 versus 2008-2016 that calls for intervention in prevention (Che et al., 2021). Particularly, the following two populations have limited thermoregulatory reserve and are exacerbated by vascular responses to cold: the elderly (Li et al., 2023; Tochihara et al., 2021) and subjects with preexisting CVD (Tochihara et al., 2021).

In the larger picture of global warming epidemiology, cardiovascular hazard from excessive heat temperatures, conveyed via endothelial dysfunction, acute changes in blood viscosity, and impaired pharmacokinetics, will require drastic public health adjustment interventions. Conversely, ensuring adequate heating remains a critical intervention to reduce cold-related mortality (Achebak et al., 2024; Gostimirovic et al., 2020).

#### 5.2.2 Pharmacological mimics and molecular targets

In addition to behavioral treatments, pharmacology attempts to mimic or amplify the benefits of cold. The angiotensin receptor-neprilysin inhibitor sacubitril/valsartan, for example, has “cold-mimetic” effects in preclinical models, such as initiating postprandial lipid oxidation, adipocyte lipolysis, and weight loss, effects similar to induced BAT (Nikolic et al., 2022). Similarly, lipid metabolite 12-HEPE synthesized by BAT also acts as a “batokine” stimulating glucose uptake in insulin-sensitive tissues and is hence a potential new drug for diabetes (Leiria et al., 2019). While of attractive hypothetical synergy with environmental cold, their action and mechanisms remain experimentally untested regarding this (Nikolic et al., 2022).

Direct modulators of cold-induced injury cascades are other drugs. For instance, inhibition of the phosphatase activity of soluble epoxide hydrolase (sEH-P) promotes lipolysis, BAT thermogenesis, and basal cardiac mitochondrial function, with subsequent ischemia-reperfusion protection (Leuillier et al., 2023). Novel targets arise, such as the cold shock protein CSDE1 in hepatocytes whose downregulation increases dyslipidemia by mRNA stabilization of the LDL receptor and offering a new target for CVD prevention (Smith et al., 2022). Even the natural product ginsenoside Rc holds promise, protecting against cold-induced myocardial injury in rats through SIRT1 activation and suppression of inflammation and apoptosis (Xue et al., 2021).

#### 5.2.3 Targeted attack on critical pathways

The new molecular understanding of cold stress holds the promise of directing the way towards targeted therapy.

Cold-inducible RNA-binding protein (CIRP): CIRP is a physiological electrophysiological stabilizer of the heart, which regulates sinoatrial node cAMP by stabilization of PDE4B/4D mRNA and avoidance of tachycardia (Xie et al., 2020a). Lacking CIRP, however, generates a pro-arrhythmic state, and therefore therapeutic measures to maintain CIRP function may be a novel anti-arrhythmic modality (Xie et al., 2020b; Zhong et al., 2021).

Brown Adipose Tissue (BAT) Activation: Pharmacological activation of BAT remains an promising approach. Angiotensin-(1–7) acting via its Mas receptor induces WAT browning and thermogenesis and entrains the renin-angiotensin system into the regulation of metabolism (Vargas-Castillo et al., 2020). During bacterial sepsis, FGF21 produced in the liver is critical in preserving cardiac output and thermogenesis but by non-cold adaptation mechanisms (Huen et al., 2021). The devastating outcomes of BAT failure can be best described by heart failure, in which it leads to dysfunction by generating TMAO (Yoshida et al., 2022).

Prevention of Pathological Processes: Another logical solution is the prevention of the downstream damage effects. As a simple example, Cold-provoked cardiomyocyte ferroptosis, and direct interference with Beclin1 by genetic or pharmacological means could inhibit cardiac issues caused by cold (Yin et al., 2020). There will be very personalized risk assessment and analysis which includes physiological monitoring, information about biomechanics, and molecular biomarkers to secure people such as soldiers who will subject themselves to hostile conditions in the future (Lee et al., 2023). The initial studies concerning the use of supplements such as the use of GABA by an athlete who is supposed to train under cold conditions have yet to show any negative effects on thermoregulation but the positive ones are yet to be well established (Wang et al., 2022).

## 6 Conclusion and future perspective

**FIGURE 4:**
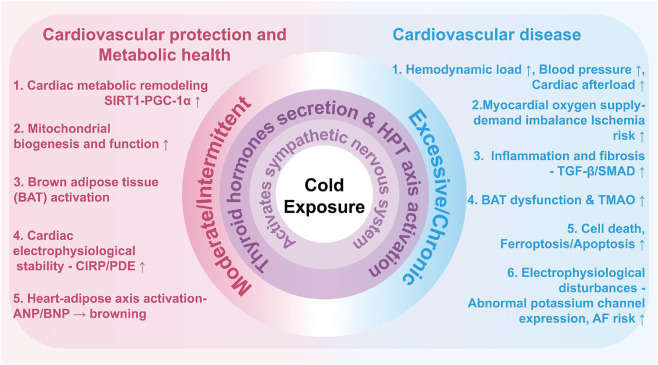
Biphasic Regulatory Network of Cold Exposure on the Cardiovascular System. Intermittent but moderate cold exposure (left pathway) prompts the activation of sympathetic nervous system and favorable activation of the hypothalamic-pituitary-thyroid (HPT) axis, which affect the systemic metabolism and tolerance of the cardiac system. This triggers a protective cascade including brown adipose tissue (BAT) thermogenesis, cardiac metabolic optimization (e.g., via SIRT1-PGC-1α), electrophysiological stabilization (mediated by CIRP), cardiac-adipose axis activation, and ultimately cardiovascular benefits. In contrast, exaggerated, chronic cold exposure (right pathway) induces sympathetic hyperactivation coupled with a maladaptive or excessive stimulation of the HPT axis, contributing to a pathologic cascade of acute hemodynamic overload, myocardial ischemia, electrophysiological dysfunction, inflammation, fibrosis, cell death, and ultimately increased risks of coronary artery disease (CAD), heart failure (HF), atrial fibrillation (AF), and hypertension (HTN). Key regulatory molecules in these opposing processes include CIRP, SIRT1-PGC-1α, and TMAO.

This review summarizes the existing findings to describe cold exposure as a example of a two-edged that has been shown to be beneficial to cardiovascular health (Figure 4). The end result, be it maladaptive pathology or salutary adaptation, is non stochastic but is the result of a critical interplay between the exposure variables (duration, intensity, pattern) and host characteristics (underlying health status, physiological reserve) (Al-Horani et al., 2021; Yang et al., 2019; Chen et al., 2024).

The physiological dualism is evident. On the one hand, mild and intermittent cold may be effective physiological stimuli to improve cardiovascular resilience by increasing brown adipose tissue functionality, optimizing metabolic functions, and refining autonomic functions, as is demonstrated by lower ischemia-reperfusion damage and lowering the pathological remodeling in preclinical animals. Conversely, to the susceptible individuals, it is a strong environmental stressor, causing a sequence of hemodynamic and metabolic disruptions leading to acute clinical events, an event supported by solid epidemiological relationships and clear pathophysiology (Ald et al., 2022; Jin et al., 2023; Zhang W. et al., 2021; Lin et al., 2020; Walther et al., 2021).

In order to go a step further with this phenomenological knowledge to mechanistic prediction and individualized application, the discipline needs to initiate a series of dedicated research projects. One major issue here is to unravel the actual molecular factors that regulate the balance point of cardioprotection to damage (Pernes et al., 2021). This will require a transition to interventional studies, including using a tool to manipulate key candidates, e.g., CIRP, SIRT1 and autophagic machinery components, with a tool like a tissue-specific gene editing tool in an animal model to control variables much more precisely (Ushio and Eto, 2021; Herrmann et al., 2024; Zhang L. et al., 2021).

At the same time, the most promising idea of cold adaptation has to be carefully experimented on human populations. Randomized controlled trials are schematically required to assess the effectiveness and the safety of a specific cold modality (e.g., the efficacy of controlled ambient acclimation, whole-body cryostimulation) (Bongers et al., 2019) in the nearest future. These experiments have to include well-characterized subpopulations (e.g., patients with heart failure with preserved ejection fraction, or with stable coronary artery disease) and capture long-term outcomes such as traditional cardiovascular biomarkers, brown adipose tissue activity (where using ^18^F-FDG-PET/CT), cardiac function, and quality of life and long-term follow-up to define sustainability and safety.

In accordance with this, the revolution of individual medicine demands creation of machinery to foretell personal susceptibility to cold pressure. One such promising strategy is to combine real-time wearable and multi-omics profiling of biobanked samples to create dynamic biomarker panels and AI. This may help to build a customized cold stress index which would help to pre-empt high risks people when cold spells occur (Thibonnier et al., 2022).

Lastly, with the backdrop of changing climatic conditions, rising incidents of extreme temperatures, the study should measure the future cardiovascular disease burden due to changing patterns of cold and model the cost-effectiveness of different public health measures. This involves maximizing early-warning systems, assessing the cardiovascular benefits of insulating housing structures and energy-assistance schemes and coming up with specific guidelines to secure vulnerable groups like the elderly and pre-existing cardiovascular patients (De Vita et al., 2024; Abrignani et al., 2022).

Conclusively, cold with its complimentary and contrasting edge certainly poses a daunting challenge and a spectacular opportunity. The future in this field does not just depend on observing its impact but rather using this intense stimulus of the environment. We can hope to change our relationship with cold, not to passively alleviate an environmental hazard but to wisely enlist the help of cold in the achievement of cardiovascular health, by understanding its underlying mechanisms, test designed therapeutic measures and making informed policies of public health.

## Glossary

AAD: Acute Aortic Dissection
ACS: Acute Coronary Syndromes
AMI: Acute Myocardial Infarction
ANP/BNP: Atrial Natriuretic Peptide/Brain Natriuretic Peptide
Ang-(1–7): Angiotensin-(1–7)
ATGL: Adipose Triglyceride Lipase
BAT: Brown Adipose Tissue
CAD: Coronary Artery Disease
CIRP: Cold-Inducible RNA-Binding Protein
CKD: Chronic Kidney Disease
CPT: Cold Pressor Test
CVD: Cardiovascular Disease
CWI: Cold-Water Immersion
Cx43: Connexin 43
DM-C: 2,5-Dimethylcelecoxib
eCIRP: extracellular Cold-Inducible RNA-Binding Protein
ECM: Extracellular Matrix
HAC: High-Altitude and Cold
HF: Heart Failure | High-Frequency (in HRV)
HRV: Heart Rate Variability
LF/HF: Low-Frequency/High-Frequency ratio (in HRV)
LQTS: Long QT Syndrome
MAPK: Mitogen-Activated Protein Kinase
MCA: Moderate Cold Acclimation
MI: Myocardial Infarction
OHCA: Out-of-Hospital Cardiac Arrest
PGC-1α: Peroxisome Proliferator-Activated Receptor Gamma Coactivator 1-alpha
RAS: Renin-Angiotensin System
RMSSD: Root Mean Square of Successive Differences (in HRV)
RPP: Rate-Pressure Product
SCD: Sudden Cardiac Death
SIRT1: Sirtuin 1
SK3: Small-Conductance Calcium-Activated Potassium Channel (subtype 3)
SNS: Sympathetic Nervous System
SYNTAX: Synergy between PCI with TAXUS and Cardiac Surgery (score)
TAG: Triacylglycerol
TCN: Temperature Change between Neighbouring days
TGF-β1: Transforming Growth Factor Beta 1
TMAO: Trimethylamine N-oxide
TRPM8: Transient Receptor Potential Cation Channel Subfamily M Member 8
UCP1: Uncoupling Protein 1
WAT: White Adipose Tissue
WBC: Whole-Body Cryostimulation
